# Association of retinoids, retinoic acid receptors and epigenetics in breast cancer

**DOI:** 10.1038/s41388-026-03699-8

**Published:** 2026-02-20

**Authors:** Łukasz Szymański, Tino Schenk, Michał Ławiński, Annamaria Brioli, Arthur Zelent

**Affiliations:** 1 Department of Molecular Biology, Institute of Genetics and Animal Biotechnology, Polish Academy of Sciences, Magdalenka, Poland; 2 Department of Hematology/Oncology, Clinic of Internal Medicine II, Jena University Hospital, Jena, Germany; 3 Institute of Molecular Cell Biology, Center for Molecular Biomedicine Jena (CMB), Jena University Hospital, Jena, Germany; 4 Institute of Genetics and Animal Biotechnology, Polish Academy of Sciences, Magdalenka, Poland; 5 Hematology, Hemostasis, Oncology and Stem Cell Transplantation, Hannover Medical School (MHH), Hanover, Germany

**Keywords:** Breast cancer, Epigenetics, Epigenetics

## Abstract

Retinoic acid signaling, mediated through its receptors (RARs and RXRs), plays a fundamental role in regulating cell differentiation, proliferation, and apoptosis. While well established in hematologic malignancies, particularly acute promyelocytic leukemia, its therapeutic potential in breast cancer remains underexplored. Emerging evidence has identified aberrant epigenetic regulation of retinoic acid receptors as a central mechanism of resistance to retinoic acid. This review integrates recent advances in epigenetic control, receptor biology, and translational studies to re-evaluate the therapeutic potential of retinoic acid in breast cancer. Among the many factors that influence retinoic acid signaling are reduced receptor expression and altered intracellular delivery of retinoic acid. Promoter hypermethylation and histone deacetylation silence *RARβ2* and disrupt canonical retinoic acid transcriptional networks, while imbalanced intracellular routing via CRABP2 and FABP5 and subtype-specific expression of RAR isoforms further determine therapeutic outcomes. Luminal tumors with preserved RARα and CRABP2 expression display strong retinoic acid sensitivity, in contrast to HER2-enriched and triple-negative subtypes, where *MYC*-driven *CRABP2* suppression and DNA hypermethylation confer retinoid resistance. Epigenetic therapies using DNMT or HDAC inhibitors can restore RARβ2 expression and resensitize tumors. Combination regimens such as retinoic acid with entinostat and doxorubicin achieve potent antitumor synergy in preclinical models. Retinoic acid also remodels the tumor microenvironment by modulating angiogenesis, fibroblast activation, and immune responses, although stromal RARβ signaling can paradoxically promote tumor progression. Early clinical trials lacked biomarker stratification and were limited by unfavorable pharmacokinetics, likely obscuring therapeutic benefit. Future clinical development should focus on biomarker-driven patient stratification, pharmacological optimization, and rational combination strategies that integrate retinoids with targeted or immune-based therapies. Notably, emerging methylation-based classifiers that identify retinoid-responsive triple-negative breast cancer subsets, together with the paradoxical pro-tumorigenic effects of stromal RARβ, underscore the novelty and translational significance of integrating tumor-intrinsic and microenvironmental determinants of retinoid sensitivity. Together, these approaches may help re-establish functional retinoid signaling and realize the therapeutic potential of retinoic acid in breast cancer.

## Introduction

**Fig. 1:**
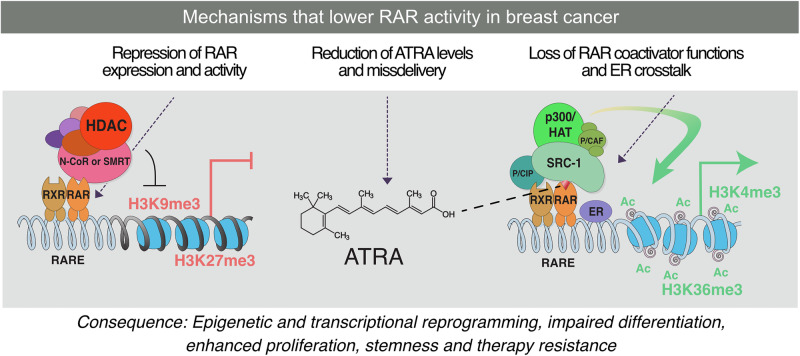
Overview of retinoic acid receptor (RAR) and retinoid X receptor (RXR) signaling and mechanisms that impair RAR activity in breast cancer. RARα, RARβ, and RARγ form obligate heterodimers with RXRα, RXRβ, or RXRγ on retinoic acid response elements (RAREs) in chromatin. Binding of all-trans retinoic acid to RAR induces co-repressor release and recruitment of co-activator complexes, including histone acetyltransferases, which promote chromatin opening and transcription of genes involved in cell-cycle arrest, differentiation, and apoptosis. In breast cancer, this pathway can be disrupted through reduced RAR expression, limited intracellular ATRA availability, altered retinoid routing by CRABP2 or FABP5, and impaired co-activator recruitment or crosstalk with the estrogen receptor. These defects prevent effective transcriptional activation and contribute to epigenetic reprogramming, enhanced proliferation, and therapeutic resistance.

Breast cancer is the most common malignancy in women worldwide. It is characterized by profound molecular heterogeneity and encompasses luminal (ER/PR + ), HER2-enriched, and basal-like/triple-negative subtypes. Each subtype has distinct biology and therapeutic vulnerabilities [1]. While alterations in the *TP53*, *PIK3CA*, and *ERBB2* genes are well-established as contributing factors, recent evidence suggests that epigenetic reprogramming through DNA methylation, histone modifications, and non-coding RNAs plays an equally important role in initiating, progressing, and developing therapeutic resistance in breast cancer [2, 3]. Breast cancer treatment has advanced significantly, yet many patients continue to experience limited responses or disease recurrence. Estrogen receptor-positive tumors represent the largest subtype and are typically managed with endocrine therapy, but resistance emerges frequently during prolonged treatment. *RARA* is an estrogen-regulated gene, and its expression is reduced when tumors adapt to anti-estrogen therapy. This reduction weakens retinoic acid-driven transcriptional programs and creates a permissive environment for endocrine resistance [4]. In this context, strategies that restore *RARA* function may complement endocrine therapy by reinforcing differentiation-associated gene networks. Triple negative breast cancers (TNBC) lack estrogen receptor, progesterone receptor, and HER2 expression, which leaves patients without effective targeted therapies. These tumors often exhibit aggressive clinical behavior. Preclinical studies show that a subset of triple-negative breast cancers remains retinoic acid responsive and undergoes growth inhibition and induction of differentiation when exposed to ATRA [5]. This suggests that retinoid-based therapies may offer targeted approaches for selected patients within this difficult-to-treat group. Together, these observations highlight the need to define how alterations in the retinoic acid pathway contribute to therapeutic resistance in estrogen receptor-positive disease and how they may support new targeted strategies in TNBC. Retinoic acid (RA) signaling is of particular interest among the pathways dysregulated by epigenetic mechanisms. The most active metabolite of vitamin A, all-trans retinoic acid (ATRA), exerts pleiotropic effects on cell differentiation, proliferation, and apoptosis by binding to members of two nuclear receptor families, the retinoic acid receptors RARα, RARβ, and RARγ and the retinoid X receptors RXRα, RXRβ, and RXRγ (Fig. 1) [6, 7]. These six receptors are encoded by distinct genes, *RARA, RARB, RARG, RXRA, RXRB*, and *RXRG*, which generate multiple isoforms through the use of alternative promoters and alternative splicing. RAR and RXR proteins form obligate heterodimers on DNA and recognize retinoic acid response elements in regulatory regions of target genes, where ligand binding triggers exchange of corepressors for coactivator complexes and transcriptional activation [8, 9]. In this heterodimeric complex, ATRA is a high-affinity ligand for RARs, whereas 9-cis-retinoic acid and synthetic rexinoids such as bexarotene preferentially activate RXRs [8, 10]. Among the *RARB* isoforms, *RARβ2* denotes the transcript initiated from the CpG-rich P2 promoter that contains a canonical retinoic acid response element and is strongly induced by RA. This *RARβ2* promoter is frequently hypermethylated and transcriptionally silenced in breast cancer, and RARβ2 loss is a central event in RA resistance [3]. In acute promyelocytic leukemia, pharmacologic doses of ATRA target oncogenic RARα fusion proteins and, in combination with arsenic trioxide or chemotherapy, overcome a differentiation block and achieve high cure rates [11]. Although the success of ATRA in acute promyelocytic leukemia is historically important for establishing retinoids as differentiation agents, this mechanism is specific to hematologic diseases. In APL, retinoic acid acts on RARα fusion proteins that block myeloid maturation. Breast cancers do not contain RAR fusion proteins, and retinoic acid signaling in these tumors depends on intact RARα, RARβ, and RARγ receptors and on proper regulation of ligand availability, receptor expression, and nuclear receptor crosstalk [12]. As a result, the therapeutic principles learned from APL cannot be directly applied to breast cancer, although the general concept that pharmacologic retinoids can reprogram transcription remains relevant for solid tumors. In solid tumors, such as breast cancer, RARα, RARβ, and RARγ are not rearranged but are frequently downregulated or epigenetically silenced, and disruption of RA metabolism and transport further reduces effective ligand availability at the receptor [12]. These alterations help explain why, despite promising preclinical results, translation of ATRA into breast cancer therapy has been challenging [13, 14]. Beyond partnering with RARs, RXRs also function as obligatory heterodimeric partners for several other nuclear receptors, including the peroxisome proliferator-activated receptors, thyroid hormone receptors, liver X receptors, and the vitamin D receptor [8, 10]. These RXR-containing heterodimers have independent roles in breast cancer biology. Activation of peroxisome proliferator-activated receptor gamma (PPARγ) by natural and synthetic ligands reduces proliferation, motility, and invasion of breast cancer cells and favorably modulates the tumor microenvironment [15, 16]. Vitamin D signaling through the vitamin D receptor regulates mammary epithelial proliferation, differentiation, and stemness, and higher vitamin D receptor (VDR) expression and vitamin D activity are associated with more favorable breast cancer features and outcomes [17, 18]. Thyroid hormone receptors and the liver X receptors also influence metabolic control and proliferation in breast cancer, and their activity is shaped by RXR availability. Together, these interactions highlight that RXR biology extends beyond classic RAR-mediated signaling and contributes additional layers of regulation that intersect with breast cancer progression [19, 20].

Differences in retinoic acid sensitivity across breast cancer subtypes have been linked to variations in RAR expression and retinoic acid signaling competence. Luminal tumors generally retain higher RARα expression and more complete activation of retinoic acid-induced gene programs, which contributes to their greater responsiveness to ATRA [5]. In contrast, basal-like and HER2-enriched tumors frequently exhibit reduced expression of RARα or RARβ and show altered intracellular retinoic acid handling, which together limit the ability of ATRA to activate tumor suppressive transcriptional responses [7]. These molecular differences help explain the limited responsiveness of basal-like and HER2-enriched cell lines to retinoic acid. Because *RARA* is an estrogen-regulated gene, its expression is highest in luminal cells and lower in subtypes that lack estrogen receptor activity, which further contributes to subtype-specific variation in retinoic acid sensitivity [4].

RAR-dependent signaling regulates multiple tumor suppressive programs in breast cancer. Ligand-activated RARα, RARβ, and RARγ bind retinoic acid response elements and activate genes that promote cell cycle arrest, apoptosis, and epithelial differentiation. RAR activation induces transcription of *CDKN1A* and other regulators that slow G1 to S cell cycle progression and enhance expression of pro-apoptotic mediators, including BAX, thereby sensitizing cancer cells to programmed cell death [10]. RAR signaling also suppresses genes that maintain a stem-like or basal-like phenotype and promotes restoration of epithelial features through regulation of cytokeratin expression and cell adhesion genes [5]. Together, these transcriptional programs contribute to growth inhibition and reduced invasive behavior. These mechanisms reflect the canonical, RARE-dependent actions of RARs that directly reprogram proliferation, differentiation, and survival pathways in breast cancer.

Beyond classical RARE-mediated transcription, retinoic acid can also regulate gene expression through RARE-independent mechanisms. These include interactions of RARs with other transcription factors, such as the Estrogen Receptor and with epigenetic modifiers [4]. Interferon regulatory factor 1 (IRF1) has emerged as a pivotal mediator of these noncanonical pathways. IRF1 directly modulates genes involved in DNA repair, cell-cycle control, and antigen presentation, thereby amplifying retinoid tumor-suppressive functions [21]. Additionally, retinoids induce the TNF-related apoptosis-inducing ligand (TRAIL) death signaling pathway through upregulation of death receptor 5 (DR5), enhancing selective apoptosis of tumor cells and inhibiting metastasis [22, 23]. Together, these canonical and noncanonical pathways demonstrate that RARs act not only as differentiation-inducing factors but as broad transcriptional regulators that integrate apoptotic, immune-related, and epigenetic programs to suppress breast cancer progression.

This review explores the regulatory landscape of retinoid signaling in breast cancer, focusing on how epigenetic alterations, receptor isoform expression, and intracellular retinoid trafficking collectively determine therapeutic outcomes. We focus primarily on RAR-based signaling in breast cancer, while acknowledging that RXR crosstalk with PPARs, VDR, and other partners provides an additional layer of complexity that may also influence response to retinoid-based therapies. We examine emerging mechanisms of resistance and the potential to restore retinoid responsiveness through epigenetic reprogramming and rational combination strategies involving histone deacetylase (HDAC) or DNA methyltransferase (DNMT) inhibitors. By integrating molecular, epigenetic, and translational insights, we aim to reassess the therapeutic relevance of retinoids in breast cancer and to outline biomarker-guided and next-generation approaches to overcome retinoid resistance.

## Epigenetics in breast cancer

Breast cancer pathogenesis is driven not only by genetic mutations but also by extensive epigenetic reprogramming. Epigenetic alterations, heritable yet reversible changes in gene expression without modifications in DNA sequence, reshape the chromatin landscape and regulate activities of oncogenes, tumor suppressors, and signaling pathways essential for tumor progression and therapeutic response [2, 24]. These chromatin-based changes arise early in tumor evolution and accumulate during progression, providing malignant cells with transcriptional plasticity and the ability to rapidly rewire gene expression in response to therapeutic pressure. In addition to their biological significance, epigenetic profiles may serve as biomarkers for subtype classification and predictors of therapeutic response, particularly regarding retinoid sensitivity and clinical outcome. However, most mechanistic insights derive from cell-line models and require validation in patient-derived and clinical systems.

Among the most prominent epigenetic changes in breast cancer is aberrant DNA methylation. Promoter hypermethylation frequently silences tumor suppressor genes such as BRCA1, CDKN2A/p16, and RASSF1A, effectively mimicking loss-of-function mutations and disrupting DNA repair, checkpoint control, and apoptotic pathways [3]. Conversely, global hypomethylation destabilizes the genome and activates oncogenes, fueling cancer progression and metastasis. Methylation patterns also differ across molecular subtypes, with basal-like and triple-negative breast cancers showing more extensive hypermethylation than luminal tumors, suggesting that epigenetic signatures could guide subtype-specific therapeutic approaches and help identify tumors with high transcriptional plasticity [25].

Histone modifications represent a second layer of epigenetic deregulation. In breast cancer, various overactive histone deacetylases (HDACs) frequently silence tumor suppressor genes, while aberrant methylation of H3K9 and H3K27 reinforces repressive chromatin states [26]. Because histone modifications actively regulate transcription and are reversible, targeting histone-modifying enzymes offers a promising strategy to restore differentiation programs in resistant tumors [27].

Non-coding RNAs further integrate into this network of control. MicroRNAs such as miR-30a act as tumor suppressors, while oncogenic miRNAs target mediators of differentiation signaling, shaping therapy response [28]. Long non-coding RNAs serve as scaffolds for chromatin-modifying complexes, reinforcing oncogenic states [29]. Beyond RA-specific interactions, dysregulated ncRNAs intersect with broader oncogenic pathways including phosphoinositide 3-kinase (PI3K)/ protein kinase B (AKT) and Janus kinase (JAK)/signal transducer and activator of transcription (STAT), amplifying their influence on tumor biology 30]. The emergence of circulating miRNAs as minimally invasive biomarkers highlights their translational potential for prognosis and therapeutic stratification.

The cumulative impact of genetic mutations and epigenetic reprogramming is a profoundly altered gene expression landscape in breast cancer [2]. Tumor suppressor silencing (e.g., *TP53, RB1, BRCA1*) removes essential checkpoints, while oncogene activation (*MYC*, RAS family genes, *CCND1*) promotes proliferative and survival advantages [2]. Epigenetic reprogramming also drives epithelial-to-mesenchymal transition (EMT), facilitating metastasis through loss of polarity, altered adhesion, and cytoskeletal reorganization [31]. Moreover, genes controlling immune regulation and drug efflux pumps are epigenetically modulated, further shaping therapeutic resistance and the tumor microenvironment [7, 31]. High-throughput epigenomic profiling now allows comprehensive mapping of these changes, providing a basis for precision medicine approaches that integrate subtype-specific vulnerabilities with targeted epigenetic and retinoid therapies.

## Preclinical evidence of epigenetic regulation of retinoic acid receptors in breast cancer

**Fig. 2:**
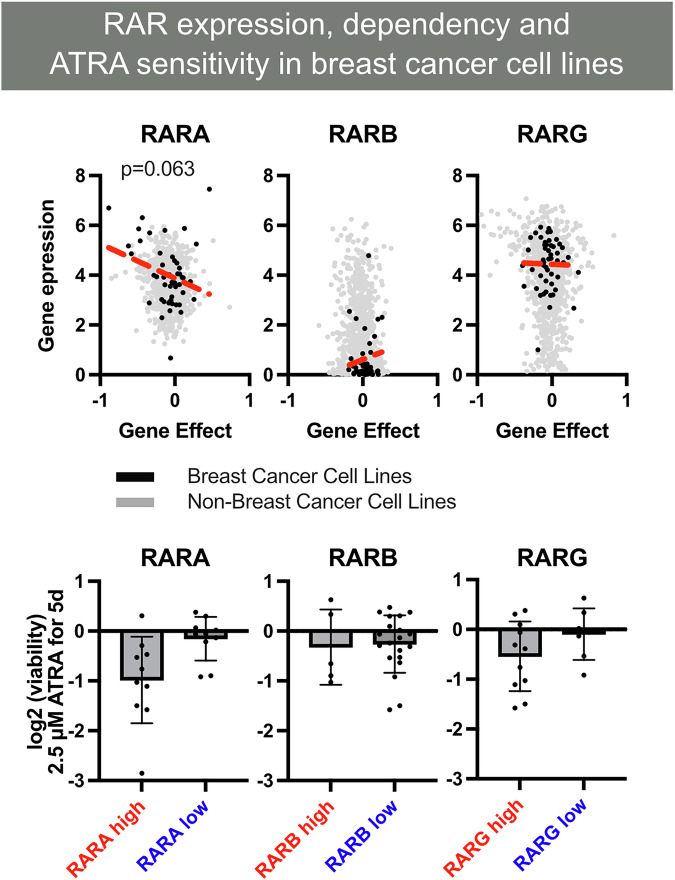
RAR isoform expression, functional dependency, and ATRA sensitivity in breast cancer cell lines. Upper panel: relationship between *RARA*, *RARB*, and *RARG* mRNA expression and CRISPR-Cas9 gene dependency scores (DepMap CERES gene effect). Negative dependency scores indicate that loss of a gene reduces cell viability, reflecting functional reliance on that receptor. Breast cancer cell lines with higher RARA or RARG expression display greater dependency on these receptors, suggesting that they retain RAR-driven transcriptional programs. Lower panel: ATRA treatment selectively reduces viability in cell lines with high RARA and RARG expression, consistent with subtype-specific retinoic acid responsiveness. Intact RAR signaling capacity is a key determinant of ATRA sensitivity in breast cancer.

**Fig. 3:**
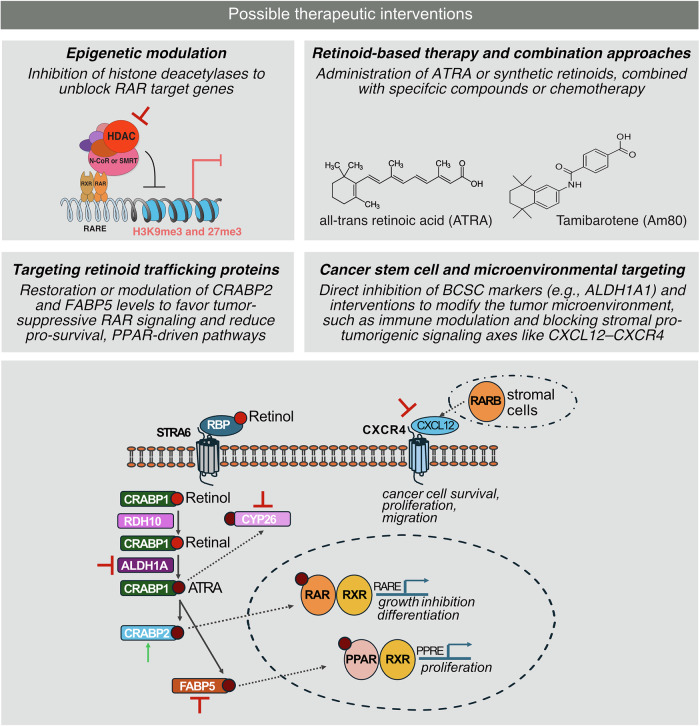
Therapeutic strategies to restore retinoic acid receptor (RAR) signaling in breast cancer. Multiple mechanisms disrupt RAR activity in breast cancer, and several therapeutic approaches can reestablish effective retinoid signaling. (i) Epigenetic modulation: Histone deacetylase (HDAC) inhibitors and DNA methyltransferase inhibitors relieve promoter repression at RAR target genes, particularly RARβ2, restoring ligand-dependent transcription. (ii) Pharmacologic restoration of ligand availability: Administration of all-trans retinoic acid or synthetic retinoids such as tamibarotene can re-engage RAR signaling, especially when combined with chemotherapy or pathway-targeted agents that enhance differentiation responses. (iii) Targeting intracellular retinoid routing: Increasing CRABP2 or reducing FABP5 shifts ATRA delivery toward RARα and away from PPARβ/δ, promoting tumor-suppressive differentiation pathways. (iv) Disrupting tumor-initiating and stromal resistance programs: Inhibition of ALDH1A1 reduces retinoid-driven stemness, while blockade of CXCL12–CXCR4 signaling counteracts stromal-driven resistance. Together, these strategies highlight multiple points of intervention capable of restoring RAR function in retinoid-refractory breast cancers.

Among the retinoic acid receptors, RARβ2 has been most consistently implicated as a tumor suppressor in breast cancer. Its expression is frequently lost or downregulated in invasive tumors, not by genetic mutation but through epigenetic silencing (Figs. 2 and 3). *RARβ2* silencing occurs through a “dual-lock” mechanism: promoter hypermethylation, reported in 10-42% of breast tumors and up to 80% in some cohorts, and repressive histone modifications when promoters remain unmethylated [32, 33]. This repression is strongly associated with poor prognosis and resistance to ATRA [13]. Detection of *RARβ2* methylation in circulating DNA further suggests utility as a minimally invasive biomarker [34]. Restoring RARβ2 not only reactivates RA signaling but also globally rewires transcriptional programs. RARβ2 overexpression induces tumor suppressors and metastasis inhibitors such as *NDRG1* (N-myc downstream regulated gene 1), *ST18* (suppression of tumorigenicity 18), and *TYRP1* (tyrosinase-related protein 1), while repressing adhesion and metabolic mediators, including CD164 and FABP6, and dampening AP-1 activity through competition for shared co-activators such as CBP/p300 [4, 7, 35]. These shifts explain both the antimetastatic and differentiation-inducing phenotypes observed in xenograft models, where RARβ2 expression reduced metastatic incidence from 37% in controls to 1.8% in RARβ2-expressing tumors.

Treatment with DNA demethylating agents such as 5-aza-2′-deoxycytidine or HDAC inhibitors such as entinostat restores RARβ2 expression, reactivating RA signaling in resistant breast cancer cells, inducing tumor regression in xenograft models [36–38]. These findings establish RARβ2 as both a predictive biomarker and a therapeutic target for re-sensitization strategies.

While RARβ2 repression is the most extensively studied, other RAR isoforms are also subject to epigenetic regulation. RARα, essential for luminal differentiation, is generally preserved in ER+ tumors and correlates with sensitivity to ATRA [5]. In contrast, RARγ exhibits oncogenic properties in several solid tumors, including breast cancer, where it promotes stem-like features and survival signaling [24]. Expression analyses indicate that RARα and RARγ are broadly co-expressed in most breast cancer samples, although in certain cell lines only one isoform is predominantly expressed, suggesting potential subtype- or context-dependent regulation of these receptors. Dysregulation of miR-30a, which normally constrains RARγ, enhances its oncogenic transcriptional activity and contributes to retinoid resistance. Thus, the balance of RAR isoform expression, sculpted by DNA methylation, histone modifications, and ncRNAs, dictates whether RA signaling drives differentiation and apoptosis or tumor promotion.

In addition to receptor-level changes, intracellular routing of retinoic acid strongly influences signaling outcomes. Cellular retinoic acid binding protein 2 (CRABP2) and fatty acid binding protein 5 (FABP5) are cytosolic lipid chaperones that bind ATRA and deliver it to distinct nuclear receptors. CRABP2 transports ATRA into the nucleus and presents it to RARα, which promotes transcription of genes that drive differentiation and apoptosis, whereas FABP5 delivers ATRA to PPARβ/δ and activates a pro-survival transcriptional program [39]. In mammary epithelial and breast cancer models, a high FABP5 to CRABP2 expression ratio converts ATRA responses from growth inhibition to proliferation, while genetic or pharmacologic reduction of FABP5 or enforced CRABP2 expression redirects RA toward RARα and restores tumor suppressive signaling [40]. Resistance is also influenced by loss of transcriptional coactivator recruitment to ligand-bound RARs. This is often due to disruption of the activation function 2 (AF2) helix in the ligand-binding domain, which forms the docking surface for coactivators that contain LXXLL motifs such as SRC family proteins and CBP or p300 [9]. Defects in AF2 structure or in coactivator complexes impair effective receptor activation even in the presence of ligand [39, 40]. In HER2-enriched breast cancers, MYC-mediated suppression of CRABP2 further distorts this chaperone balance and effectively disables RA–RARα signaling, an effect that can be reversed by MYC inhibition or CRABP2 re-expression [14]. Restoring CRABP2 expression or reducing FABP5 therefore redirects RA flux toward differentiation pathways. HER2-enriched tumors often combine this CRABP2 or FABP5 imbalance with insufficient RARα activity and epigenetic silencing of the RARβ2 promoter, which together severely limit retinoic acid responses [5]. Moreover, cellular retinoic acid binding protein 1 (CRABP1), which is another retinoid-binding protein that sequesters RA, adds further complexity to intracellular retinoid routing and resistance mechanisms [41].

Additionally, a truncated tumor-specific isoform, RARβ-prime, has been identified in breast cancer cells (MCF-7) and linked to impaired ATRA-responsiveness [42]. Another layer of resistance involves overexpression of Activator Protein-1 components (*JUN, FOS*), which antagonize RA effects. Uniquely, RARβ can inhibit AP-1 activity independent of ligand binding, whereas RARα and RARγ require ligand engagement for repression [43, 44].

Beyond HDACs and DNMTs, histone demethylases such as lysine-specific demethylase 1 (LSD1) have emerged as relevant regulators of RA signaling. LSD1 inhibitors prevent the decommissioning of RA-responsive enhancers and enhance RA activity, as shown in acute myeloid leukemia, where tranylcypromine can restore differentiation and sensitivity to ATRA [45]. Preliminary evidence indicates that similar sensitization mechanisms operate in breast cancer, suggesting that LSD1 blockade could complement DNMT and HDAC inhibition to restore retinoid responsiveness [46, 47].

Epigenetic regulation also extends to enzymes involved in retinoic acid biosynthesis. ALDH1A1, which is responsible for the final step in RA synthesis, is highly expressed in breast cancer stem cell (BCSC) populations (ALDH1A1^^high^/CD44^^high^) and marks aggressive, therapy-resistant clones. Retinoids, including retinoic acid and third-generation analogs such as adapalene, suppress BCSCs by inducing differentiation and apoptosis. Although high ALDH activity increases local RA production, these cells remain refractory to differentiation because of receptor silencing and imbalanced CRABP2/FABP5 routing[31, 48–50]. Notably, tamoxifen can paradoxically enhance stemness by upregulating ALDH1A1 in ERα36-positive subpopulations, thereby promoting metastasis and relapse[49]. This paradox underscores that RA synthesis alone is insufficient; correct intracellular trafficking and receptor activation are essential for therapeutic efficacy. Pharmacologic inhibition of ALDH1A1 or restoration of RARB and CRABP2 expression represents a promising approach to overcome stemness-driven resistance.

RARs themselves actively shape the epigenome. In the absence of ligand, RAR/RXR heterodimers recruit co-repressors such as NCoR/SMRT complexes, HDACs, and DNMTs, maintaining a repressed chromatin state at RA-responsive loci. Upon ligand binding, conformational changes release these repressors and recruit histone acetyltransferases (HATs) (CBP/p300, PCAF), Mediator complexes, and chromatin remodelers, enabling transcriptional activation [8, 35, 51]. When *RARB* is epigenetically silenced, this regulatory loop is disrupted, further impairing RA-mediated transcription and contributing to resistance.

In addition to its tumor-cell-intrinsic effects, ATRA profoundly remodels the tumor microenvironment. The foundational role of the breast tumor stroma was established by the identification of stromelysin-3/MMP11 as a metalloproteinase specifically expressed in stromal fibroblasts adjacent to invasive carcinomas, highlighting stromal remodeling as an active driver of tumor behavior [52]. ATRA reduces angiogenesis, alters cancer-associated fibroblast states, and modulates immune surveillance by enhancing T-cell infiltration and macrophage polarization toward antitumor phenotypes [7]. Notably, RA decreases myeloid-derived suppressor cell (MDSC) populations and promotes their maturation, alleviating immunosuppression and restoring T-cell proliferation [53–55]. In breast cancer models, ATRA synergizes with anti-VEGFR2 therapy by reducing hypoxia and MDSC expansion, thereby facilitating immune cell infiltration [56]. However, stromal RARβ activation can paradoxically promote tumorigenesis by amplifying CXCL12–CXCR4 signaling, emphasizing the need for compartment-specific pharmacodynamic monitoring [34]. These microenvironmental effects demonstrate that retinoic acid exerts a broad influence beyond tumor cells themselves, and they support the rationale for integrating retinoids with immunotherapy and stromal-targeting agents.

Finally, preclinical data highlight rational combination strategies to overcome resistance. The triple combination of entinostat, retinoic acid, and doxorubicin (EAD) induced pronounced regression of triple-negative xenografts and depletion of tumor-initiating cells [36]. Mechanistically, this synergy involves entinostat-mediated HDAC and NCOR corepressor inhibition at the *RARB* promoter, derepressing RARB expression, together with doxorubicin’s inhibition of topoisomerase II-β and RA-driven differentiation signaling. Additional combinations using valproic acid or 5-aza-2′-deoxycytidine similarly restore RARB expression and proliferation control across ERα-positive and negative breast cancers [37]. Emerging evidence that RA enhances radiation therapy and immune checkpoint blockade [57, 58] reinforces the concept that epigenetic priming, differentiation therapy, and immune reprogramming can converge for durable antitumor efficacy. Together, these findings demonstrate that retinoid resistance in breast cancer arises from interdependent epigenetic, metabolic, and microenvironmental mechanisms. Strategies that restore RARB expression, normalize CRABP2/FABP5 balance, or co-target epigenetic enzymes such as DNMTs, HDACs, and LSD1 can re-establish functional retinoic acid signaling and resensitize tumors to differentiation therapy. These regulatory layers converge to determine whether retinoic acid can activate transcriptional programs essential for differentiation and growth control. Understanding these interactions is critical for designing retinoid-based strategies that overcome resistance.

## Clinical evidence

Early clinical experience with natural retinoids in breast cancer has been limited and, overall, disappointing when retinoids were used as single agents. Across phase-I programs testing ATRA and 13-cis-retinoic acid in mixed solid tumors (including breast), dose-limiting toxicities (DLTs) and maximum tolerated doses (MTDs) were defined, but objective responses were essentially absent; in a small phase-II study in metastatic breast cancer, activity was restricted to one partial response and a few cases of stable disease [42]. To date, comprehensive overviews concur that despite the transformative success of differentiation therapy in APL, translation of retinoids to solid tumors, such as breast cancer, has not yielded durable clinical benefit [12].

The tolerability profile of oral retinoids in these studies was manageable but non-trivial: mucocutaneous effects (e.g., cheilitis, xerosis), headaches, gastrointestinal symptoms, and liver-enzyme elevations were common. ATRA DLTs were reported at 175-200 mg/m² and the MTD was ~150 mg/m²/day; for 9-cis-retinoic acid, the MTD was ~100 mg/m²/day [42]. Escalation studies with 13-cis-retinoic acid in advanced breast cancer proceeded cautiously for similar toxicity considerations [42].

Several pharmacologic factors likely blunted clinical signals: ATRA’s short plasma half-life (~45 min), poor solubility, and wide interpatient variability in exposure and metabolism (Muindi, 1992, Pharmacokinetics of oral all-trans-retinoic acid; Muindi, 1994, Interpatient variability of retinoic acid metabolism; Veal, 2013, Pharmacokinetics and metabolism of retinoids in cancer therapy), along with adaptive declines in circulating levels during chronic dosing [42]. These hurdles undermine durable target engagement in solid tumors. To address them, optimized formulations (e.g., sustained-release, lipid-based delivery) and exposure-guided dosing should be paired with pharmacodynamic readouts such as RARβ2 re-expression or RA-target gene induction in tumor biopsies [7], ideally within rational combination regimens rather than monotherapy.

Clinically, the retinoid axis is “druggable,” as illustrated by RXR-selective bexarotene activity in cutaneous T-cell lymphoma [59], yet this has not translated into approved indications for breast cancer [42]. Taken together with the APL paradigm, these data suggest that retinoid effectiveness depends on disease context, pharmacology, and the presence of a permissive molecular program.

Retinoids have also been evaluated for their potential to prevent breast cancer development and to reduce the risk of recurrence. Fenretinide, a synthetic retinoid with an established safety profile, demonstrated a reduction in the incidence of contralateral breast cancer and showed evidence of long-term protective effects in premenopausal women in a randomized prevention trial [60]. More recently, renewed interest has focused on fenretinide as a preventive agent for women with hereditary or biologically defined high risk, where modulation of retinoid signaling and lipid metabolism may contribute to decreased susceptibility to new primary tumors [61]. These findings indicate that retinoid-based approaches may have value not only in therapeutic settings but also in prevention contexts for selected patient groups.

Importantly, translational work now offers concrete guidance for biomarker-driven clinical designs in breast cancer. In luminal disease, higher RARα expression and a luminal transcriptional program associate with ATRA sensitivity, nominating ER-positive cohorts for enrichment in future clinical trials of ATRA-based therapies. In triple-negative breast cancers, a DNA-methylation signature predicted response to ATRA, pointing to epigenetic stratifiers that could prospectively select patients [13]. Early clinical programs did not incorporate such molecular selection, likely diluting any benefit. Rational incorporation of these biomarkers, along with careful attention to pharmacokinetics and co-administered epigenetic or pathway-targeted treatments, represents a credible path for renewed clinical testing in subtype-defined settings [41, 62].

Retinoid combinations have advanced clinically in other solid tumors, underscoring the feasibility of modern trial designs that could be adapted to breast cancer. For example, in pancreatic cancer, a phase-I study repurposed ATRA as a stromal-targeting agent alongside gemcitabine/nab-paclitaxel within an adaptive schema, with evidence of stromal normalization, exemplifying mechanism-guided combination therapy [51]. Such combinatorial approaches could inspire analogous strategies in breast cancer. Given accumulating preclinical data supporting synergy with endocrine therapy, chemotherapy, or epigenetic agents in breast models, the next wave of trials should prospectively be enriched by RAR pathway biomarkers and epigenetic states [5]. Formulation and pharmacokinetic optimizations will also be critical to maintain on-target exposure.

## Contextual regulation of retinoic acid signaling in breast cancer

A central controversy is that RA can be either tumor-suppressive or pro-survival depending on intracellular chaperoning and receptor usage. As stated above, intracellular routing of ATRA determines its biological outcome: CRABP2 directs signaling through RARα to induce differentiation and apoptosis, whereas FABP5 favors PPARβ/δ-mediated pro-survival pathways [39]. Alongside this FABP5/CRABP2 balance, the epigenetic functionality of RARα further dictates response, as impaired RARα-dependent transcription can redirect RA signaling toward non-transcriptional, pro-invasive effects [63]. This dual regulatory mechanism provides a coherent explanation for the context-dependent, sometimes paradoxical actions of retinoic acid.

The long-held view of RARβ as a tumor suppressor is challenged by stromal genetics. In an ErbB2 (Neu)-driven mouse model, stromal Rarb deletion *reduced* mammary tumorigenesis, with decreased angiogenesis, fewer myofibroblasts, diminished inflammatory-cell recruitment, and attenuation of the *CXCL12-CXCR4* axis [34]. These data suggest that compartment-specific RARβ functions can be protumorigenic in stroma while tumor-suppressive in epithelium, complicating therapeutic expectations for pan-tissue RA activation. Moreover, it underscores the importance of compartment-resolved pharmacodynamic monitoring, including immune phenotyping, stromal signatures, and angiogenic markers-to ensure that retinoid-based therapies reinforce anti-tumor immunity rather than inadvertently promoting protumorigenic programs.

Crosstalk with the estrogen receptor (ER) pathway adds further context dependence. Genome-wide studies show extensive co-occupancy and antagonism between RARs and ERα at shared regulatory regions, providing a mechanism for RA to counter estrogen-driven programmes in ER+ disease [4]. Reciprocally, ER can shape RAR chromatin binding. Mechanistic work indicates that RARα can be required for efficient ER-mediated transcription and proliferation, revealing cooperative as well as antagonistic modes that vary with ligand conditions and cofactors [64]. In clinical cohorts, high intratumoral RARα associates with shorter relapse‑free survival among ER+ patients treated with adjuvant tamoxifen, and tamoxifen‑resistant cells exhibit heightened sensitivity to RARα ligands, indicating that RARα may serve both as a predictive marker of endocrine resistance and as a therapeutic target in the resistant state [65]. These nuances imply that RA may modulate endocrine responsiveness in ways that depend on timing, dose, and chromatin state.

Another recurrent issue is why early trials underperformed despite strong positive laboratory signals. Two factors are prominent: (i) pharmacology: short half-life, variable exposure, and adaptive declines in circulating levels with chronic dosing; and (ii) lack of biomarker selection: retinoids were tested in unselected populations, likely diluting benefit. Contemporary data identify biomarkers for enrichment: luminal/ER+ tumors with higher RARα expression show greater ATRA sensitivity [5], and TNBC methylation signatures can prospectively predict ATRA response in patient-derived xenografts [13]. These insights were not incorporated into clinical trials but now support biomarker-guided studies. In HER2-enriched disease, *RARA* amplification does not guarantee responsiveness, as resistance depends on MYC-driven suppression of CRABP2 [14]. Depleting MYC restores RA sensitivity and improves trastuzumab efficacy, identifying a biomarker triad of *HER2/RARA* co-amplification, low MYC, and high CRABP2 for patient selection.

Moreover, emerging data underscore that breast cancer heterogeneity also varies across racial and ethnic groups, with distinct tumor biology, treatment access, and outcomes observed among Hispanic Black, Hispanic White, and non-Hispanic populations. Integrating ancestry-informed and sociodemographic variables into retinoic acid–based biomarker models will be essential for equitable therapeutic translation [66]. Preliminary population-level epigenetic analyses demonstrate ancestry-associated differences in DNA methylation in breast cancer patients (e.g., African vs European ancestry) and suggest potential variation in chromatin accessibility. Although direct data linking these differences to *RARβ2* silencing and retinoic acid-pathway activity are lacking, this underscores the need to validate RA-related biomarkers across diverse ancestry cohorts [67, 68].

Finally, the tumor microenvironment remains incompletely understood. While preclinical studies show that ATRA can reprogram the niche, reducing angiogenesis, modulating fibroblasts, and influencing antitumor immunity, human data defining dose, schedule, and the immune correlates of benefit are sparse[7]. Given the stromal RARβ paradox, compartment-resolved pharmacodynamic readouts (immune phenotyping, CAF states, chemokine axes) should be embedded in upcoming trials to avoid inadvertently amplifying pro-tumorigenic stromal programs.

## Future directions for biomarker-guided combination trial design

Looking forward, the clinical translation of retinoids in breast cancer will depend on the careful implementation of biomarker-driven therapeutic strategies. In ER-positive tumors, particularly those with a luminal phenotype, high expression of RARα strongly correlates with ATRA-responsiveness and growth inhibition, making this subgroup a rational candidate for future trials [5]. In TNBC, a CpG methylation signature that distinguishes ATRA-sensitive from resistant tumors in both cell lines and patient-derived xenografts offers a promising predictive biomarker that could guide patient selection [13]. A genome‑wide study identified >1400 differentially methylated CpG sites that stratified TNBC cell lines by ATRA response and prospectively predicted sensitivity in patient‑derived xenografts, projecting that ~17% of TNBC cases could benefit from ATRA‑based therapy [13]. Post‑translational control of RARα adds a complementary layer of resistance in TNBC. Constitutive Ser77 phosphorylation of RARα, mediated by CDK7, correlates with RA insensitivity. Conversely, a phospho‑deficient RARα‑S77A mutant suppresses TNBC growth in vitro and in vivo by inducing apoptosis, cell‑cycle arrest, and cytotoxic autophagy, while up‑regulating miR‑3074‑5p to repress DHRS3. Notably, CDK7 inhibition (THZ1) reduces RARα‑S77 phosphorylation, down‑regulates DHRS3, and inhibits TNBC proliferation, highlighting a CDK7–RARα‑S77–miR‑3074‑5p/DHRS3 axis to bypass ligand resistance [69]. Together, these biomarker studies indicate that retinoid sensitivity in TNBC is determined by both epigenetic signatures and post-translational control of RARα, highlighting clear avenues for molecularly guided patient selection.

Similarly, in HER2-enriched tumors, *HER2/RARA* co-amplification alone does not guarantee sensitivity; rather, low MYC activity and sufficient CRABP2 expression are also required. Preclinical studies show that MYC-mediated suppression of CRABP2 disables RARα signaling and drives resistance, while MYC depletion restores responsiveness and synergizes with trastuzumab, underscoring the importance of this biomarker triad for clinical stratification [14]. These findings demonstrate that retinoid response in HER2-enriched tumors depends on a coordinated molecular profile, and not on *HER2/RARA* co-amplification alone.

A further determinant of retinoid efficacy lies in the routing of ATRA within the cell. When CRABP2 predominates, ATRA is delivered to RARα, driving differentiation and apoptosis, whereas high FABP5 levels divert signaling toward PPARβ/δ, with pro-survival consequences [39, 40]. A complementary barrier is enzymatic inactivation; TNBC specimens frequently express CYP26A1 (and related CYPs) that catabolize ATRA, arguing for routine assessment of RA‑catabolic enzymes in biomarker panels and for strategies that maintain intratumoral RA exposure [70]. These observations underscore that intracellular routing and RA metabolism must be considered jointly with receptor expression to reliably predict retinoid responsiveness.

Given that epigenetic silencing often underlies retinoid resistance, another key avenue for future development is rational combination therapy. In particular, priming with DNA methyltransferase inhibitors to demethylate the *RARβ2* promoter or HDAC inhibitors to restore acetylation can re-establish retinoid responsiveness [36, 37]. In TNBC xenografts, a combination of entinostat, ATRA, and doxorubicin produced significant regression of tumors and depletion of tumor-initiating cells, illustrating how epigenetic reprogramming can convert resistant phenotypes into ATRA-sensitive states [36]. In ER-positive tumors, combining ATRA with endocrine therapy is especially appealing, since genome-wide analyses show that RARα and ERα exhibit both antagonistic and cooperative binding at shared chromatin sites, suggesting a context-dependent interplay that could be therapeutically leveraged [4]. For HER2-enriched disease, biomarker-selected patients with low MYC and high CRABP2 may benefit from combinations of ATRA with HER2-targeted agents, with the additional option of MYC inhibition or CRABP2 restoration to overcome resistance [14]. Collectively, these studies show that epigenetic priming and rationally paired combination therapies can overcome multiple layers of retinoid resistance across molecular subtypes.

Emerging evidence also highlights the potential of retinoid-immunotherapy combinations. By reducing myeloid-derived suppressor cells, enhancing T-cell and macrophage activity, and alleviating hypoxia-driven immune evasion, ATRA can reprogram the tumor microenvironment to favor anti-tumor immunity [7, 54]. These effects provide a mechanistic rationale for testing ATRA alongside immune checkpoint blockade or anti-angiogenic therapies, particularly in triple-negative and HER2-enriched subtypes where immune escape remains a key driver of resistance. These microenvironmental effects suggest that pairing retinoids with immunotherapy may unlock durable responses in retinoid-refractory subtypes.

Evidence from acute myeloid leukemia (AML) further underscores the importance of RARα dosage and epigenetic context in shaping retinoid responses. In subsets of AML, a RARα super-enhancer drives very high RARα expression, which is associated with heightened sensitivity to ATRA and the RARα-specific agonist Am80 (tamibarotene) [71]. While initial reports suggested preferential activity of Am80, more recent preclinical evidence shows that ATRA and Am80 perform comparably. Instead, the most robust differentiation occurs when retinoids are combined with epigenetic modulators such as LSD1 and GCN5 inhibitors, which sustain enhancer activity and amplify retinoid signaling [45, 72]. Although the role of super-enhancer-mediated RARα overexpression in solid tumors remains largely unexplored, it represents a compelling avenue for future investigation. These insights illustrate how enhancer-driven RARα dependency and epigenetic co-targeting dictate therapeutic outcomes in AML, and they suggest that a similar biomarker-guided framework could be applied to breast cancer, particularly in subsets with *RARA* amplification or hyperactivation. Although the underlying mechanisms differ fundamentally from those in solid tumors, these AML data reinforce the broader principle that receptor abundance and chromatin state strongly shape retinoid responses. This conceptual framework can inform breast cancer–specific biomarker development without implying mechanistic equivalence between the two diseases.

While these biomarker-driven strategies are very clear, pharmacological challenges remain. ATRA is limited by its short half-life, variable plasma exposure, and adaptive declines during chronic administration, which undermine durable target engagement [42]. To address these limitations, future trials should incorporate exposure-guided dosing and explore novel formulations, including sustained-release or lipid-based systems, designed to improve pharmacokinetics. Importantly, such approaches should be paired with pharmacodynamic endpoints, such as induction of RA-target gene expression or re-expression of *RARβ2/α2* in tumor biopsies, to confirm on-target activity [7]. Pharmacokinetic improvements and pharmacodynamic confirmation will be essential to achieve reliable retinoid exposure and durable receptor engagement.

Equally critical is the evaluation of microenvironmental effects, as RA activity extends beyond tumor cells. ATRA has been shown to modulate angiogenesis, fibroblast activation, and immune responses, particularly in T cells and macrophages [7]. However, as mentioned earlier, stromal RARβ can paradoxically promote tumorigenesis by amplifying CXCL12-CXCR4 signaling, illustrating the compartment and context-specific complexity of RA action [34]. These observations highlight the need for compartment-resolved pharmacodynamic monitoring, including assessments of cancer-associated fibroblasts, angiogenic markers, and immune phenotypes, to ensure that RA-based interventions favor anti-tumor rather than pro-tumor outcomes [73]. These findings also show that future retinoid trials must incorporate stromal and immune monitoring to ensure that therapeutic effects are beneficial across tumor compartments.

Finally, trial design will be crucial to harness these insights effectively. Adaptive, multi-arm clinical architectures could enable parallel testing of retinoids in biomarker-defined cohorts, such as luminal tumors with high RARα expression, TNBC enriched by methylation-based classifiers, and *HER2/RARA* co-amplified subsets with low MYC and high CRABP2. Each trial arm should prespecify pharmacodynamic success criteria, including re-expression of RARβ2 after epigenetic priming, favorable shifts in the FABP5/CRABP2 ratio, induction of RA-target genes, and depletion of stem-like ALDH1A1^^high^/CD44^^high^ populations [13, 31, 39]. Alongside efficacy, vigilant safety monitoring will remain essential, particularly for the well-recognized mucocutaneous and hepatic toxicities of retinoids, as well as potential interactions with endocrine therapies.

Together, these approaches, anchored in biomarker-guided enrichment, mechanistic stratification, rational combinations, pharmacological optimization, and adaptive trial designs, provide a roadmap for repositioning retinoids as precision agents in breast cancer therapy. In parallel, integration of retinoids with immunotherapy strategies should be prioritized. By reshaping the tumor immune microenvironment, ATRA has the potential to synergize with immune checkpoint inhibitors and anti-angiogenic agents, opening a path toward durable responses in subtypes where immune escape is a dominant feature [7, 56].
